# Signatures of Selection and Genomic Diversity of Muskellunge (*Esox masquinongy*) from Two Populations in North America

**DOI:** 10.3390/genes12071021

**Published:** 2021-06-30

**Authors:** Josue Chinchilla-Vargas, Jonathan R. Meerbeek, Max F. Rothschild, Francesca Bertolini

**Affiliations:** 1 Department of Animal Science, Iowa State University, Ames, IA 50011, USA; mfrothsc@iastate.edu; 2 Iowa Department of Natural Resources, Spirit Lake Fish Hatchery, Spirit Lake, IA 51360, USA; jonathan.meerbeek@dnr.iowa.gov; 3 National Institute of Aquatic Resources, Technical University of Denmark, 2800 Kgs. Lyngby, Denmark; franb@aqua.dtu.dk

**Keywords:** whole genome sequencing, Muskellunge, population genomics, Fisheries, inbreeding

## Abstract

Muskellunge (*Esox masquinongy*) is the largest and most prized game fish in North America. However, little is known about Muskellunge genetic diversity in Iowa’s propagation program. We used Whole-Genome Sequencing of 12 brooding individuals from Iowa and publicly available RAD-seq of 625 individuals from the St. Lawrence River in Canada to study the genetic differences between populations, analyze signatures of selection, and evaluate the levels of genetic diversity in both populations. Given that there is no reference genome available, reads were aligned to the genome of Pike (*Esox lucius*). Variant calling produced 7,886,471 biallelic variants for the Iowa population and 16,867 high-quality SNPs that overlap with the Canadian samples. Principal component analysis (PCA) and Admixture analyses showed a large genetic difference between Canadian and Iowan populations. Window-based pooled heterozygosity found 6 highly heterozygous windows in the Iowa population and Fst between populations found 14 windows with fixation statistic (Fst) values larger than 0.9. Canadian inbreeding rate (Froh = 0.32) appears to be higher due to the inbreeding of Iowa population (Froh = 0.03), presumably due to isolation of subpopulations. Although inbreeding does not seem to be an immediate concern for Muskellunge in Iowa, the Canadian population seems to have a high rate of inbreeding. Finally, this approach can be used to assess the long-term viability of the current management practices of Muskellunge populations across North America.

## 1. Introduction

Muskellunge (*Esox masquinongy*) is a species of freshwater fish native to North America and is the largest species of the Esocidae family. Moreover, Muskellunge is considered the most prized esocid by anglers. Originally, the species could be found in large lakes and rivers ranging from central Canada, east in the waters and branches of the Saint Lawrence River and even reaching south into Tennessee [1,2]. Distinct regional strains, each composed of multiple subpopulations have been identified in the upper Mississippi River, the Great Lakes, and the Ohio River through genetic data [3]. Due to the economic benefits associated with its reputation in the sport fishing, Muskellunge was introduced into several states in the US ranging from the Midwest to Texas and even Manitoba in Canada. The wide variety of environmental conditions of the states where Muskellunge were introduced highlights the species’ adaptability [1,2]. Although sporadic sightings of individuals have been reported in Iowa, there has been no official records of native populations in the state [1]. Currently, thanks to the effectiveness of the management and stocking practices, populations of Muskellunge can be found in several areas of North America [1,4]. While a high percentage of these populations require supplementation through periodical stocking, self-sustaining although not native populations can still be found in a number of lakes and rivers [1]. 

Muskellunge were first stocked in Iowa in the 1960s with individuals from Wisconsin that can be traced to the northern strain [5]. However previous genetic research has shown evidence of admixture in Iowa’s population [1]. Despite these findings that might point to a certain degree of genetic diversity in the population, one of the most important aspects to be considered in the design of management plans for the species is the need to maintain the genetic diversity. This objective is paramount given that the vast majority of populations in Iowa are dependent on stocking for their maintenance. Moreover, the reduced number of lakes used as sources of broodstock for Iowa’s propagation program might play a role in accelerating the loss genetic diversity as it presents an increased probability of recapturing broodstock fish each year [1,6]. 

Currently, recapture of brood stock in the Iowa populations averages 37% annually [1] and therefore introducing individuals from different genetic backgrounds or capturing broodstock from different lakes might be needed. Introducing individuals of multiple genetic backgrounds into a population can have mixed effects. When native populations are small, it is beneficial to increase genetic variance, this permits for purifying selection of deleterious variants while allowing positive selection of positive ones [7]. In turn, this limits inbreeding depression [8,9]. However, the potential negative effects of stocking include reduction of genetic diversity due to the Ryman-Laikre effect in wild populations [9,10,11], and most importantly, the loss of traits related to local adaptation [9]. 

In this research, we used whole-genome sequence from 12 broodstock individuals from Iowa (6 males and 6 females) and 625 RAD-seq individuals from Saint-Lawrence river in Canada available on SRA (Sequence Read Archive) [9]. The Canadian population is composed by approximately 10 subpopulations sampled from 22 different sites. Additionally, since both populations have no recent connection, these data provide an excellent opportunity to study the signatures of selection from two different Muskellunge populations.

Given the lack of a reference genome of Muskellunge to align sequence data, the reference genome of Northern Pike (*Esox lucius*) was used as a reference. The Northern Pike is closely related to Muskellunge and is the most frequently studied member of the Esocids [12]. Both species possess the same number of chromosomes (2*n* = 50) [13] and are capable of producing hybrids known as Tiger Muskie (Esox lucius * Esox masquinongy) which are considered valuable trophies by anglers. Nonetheless, Northern Pike is known to have a wider distribution than Muskellunge, inhabiting rivers, lakes and brackish water that range from North America to Europe and Eurasia [14,15]. This project provides the opportunity to perform a preliminary genomic comparison of these two closely related species.

## 2. Materials and Methods

### 2.1. Individuals and Sequencing

**Figure 1:**
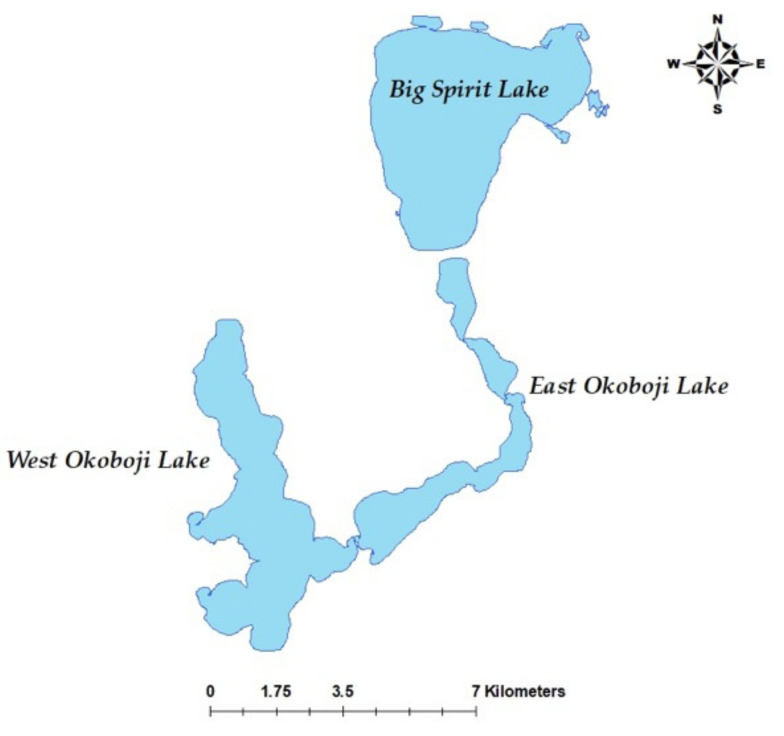
Map depicting layout of Big Spirit Lake (top) and East Okoboji Lake (center of the figure).

Iowa’s Department of Natural Resources routinely samples Muskellunge populations as part of their hatchery operations through humanely netting random individuals each spring with the number of males and females averaging 165 and 130, respectively. On average, 35 males and 15 females are used to produce the milt and eggs needed to reach production quotas of fry. As part of this standard sampling, they obtain very small fin clips to estimate the age of the fish and other projects. Whole-genome sequence was produced from samples of a total of 12 individuals from 2 lakes (6 from East Okoboji, 3 males and 3 females and 6 from–Big Spirit Lake, 3 females and 3 males) with Illumina paired-end sequencing, performed by Neogen (Lincoln, Nebraska, US). Ten of the twelve fish sampled were used that season to produce fry. As shown in Figure 1, both lakes are connected, with fish from Spirit lake being able to swim into Okoboji but not in reverse. Moreover, both lakes are stocked with fish from the same hatchery, where broodstock from the two lakes are mated and no pre-selection based on genetics is performed. Additionally, raw sequence reads of 625 samples were recovered from NCBI’s BioProject with accession number PRJNA512459 [16]. These data correspond to RAD-seq data of Muskellunge fish from different Canadian locations (detailed explanation found in Rougemont et al., 2019) [9]. Although sample size for the Iowan population is small, it has previously been shown that a small sample size allows to accurately estimate population parameters when a large number of SNPs is used [17].

### 2.2. Bioinformatics Pipeline

For all reads (Iowan and Canadian samples) read quality assessment was performed with FastQC 0.11.5 [18]. Then, reads were trimmed and filtered with Trimmomatic 0.36 [19], cropping the first 10 bases of each read, with a sliding window of 4 base pairs with a minimum quality of 20 and minimum read length of 40 bp. Given that there is no available reference genome for Muskellunge, the reference genome of a closely related fish, Northern Pike (*Esox Lucius*) version fEsoLuc1.pri was used to align the reads. Alignment was performed with BWA mem 0.7.17 [20] using default options.

SAMtools 1.10 (http://samtools.sourceforge.net, accessed on 3 February 2021) was used to remove duplicate reads and low-quality mapped reads (*q* < 20), while BCFtools 1.10.2 (http://samtools.github.io/bcftools/ bcftools.html, accessed on 3 February 2021) was used to call and filter variants. In the case of samples from Iowa, a minimum depth of 10x and quality score of at least 20 were the parameters required to retain a variant for both the Iowa and Canada populations. For both datasets, only biallelic SNPs were retained to minimize the risk of including alleles from Northern pike in downstream analyses. Additionally, monomorphic alleles were removed for downstream analyses.

### 2.3. Population Stratification Analyses

The software Admixture 1.3.0 [21] was used to estimate population stratification within both Iowa and Canadian populations as well as within each of the populations. The --cv flag was used to produce the cross-validation error and the number of subpopulations was considered accurate when the cross-validation error was lowest or at an inflection point. Additionally, principal component analyses (PCA) were performed with the flag --pca in Plink 1.9 [22] to visualize population clusterization.

### 2.4. Pooled Heterozygosity and Fst

To investigate the differences between subpopulations, Fixation Statistic (Fst) and Pooled Heterozygosity (Hp) analyses were performed for all individuals from Iowa together, males only and females only since PCA showed clustering between sexes. Hp was used to calculate whole-genome distribution of heterozygosity, averaged over 0.5 Mb sliding windows, with 50% overlapping. For each window, Hp values were calculated using the following formula [23,24]:

(1) $$\mathrm{Hp}=\frac{2\sum \mathrm{nMAJ}\sum \mathrm{nMIN}}{{\left(\sum \mathrm{nMAJ}+\sum \mathrm{nMIN}\right)}^{2}}$$

where ΣnMAJ and ΣnMIN are sums of counts of major (the most common allele for a given SNP) and minor (the least common allele for a given SNP) alleles, respectively counted at all SNPs in the window. These values were then transformed into Z scores:

(2) $$\mathrm{ZHp}\;=\left(\mathrm{Hp}-\;\mu \mathrm{Hp}\right)/\sigma \mathrm{Hp}$$

Fst score for each SNP was estimated using the --Fst flag in Plink 1.9, followed by the calculation of mean Fst values (mFst) in 500 Kbp windows with 50% overlapping using an in-house script as performed by Bertolini et al. [25]. Mean Fst scores were calculated between the populations of Iowa and Canada. 

Gene ontology analyses of the genes contained in the windows of interest for Hp and mFst analyses were performed using FishEnrichr [26,27].

### 2.5. Inbreeding and Runs of Homozygosity (ROH) 

Given the lack of pedigree information for the individuals included in this research, inbreeding coefficient (F) was estimated from runs of homozygosity (Froh) [28]. This was deemed as the most appropriate method to estimate the levels of inbreeding of both Muskellunge populations included in the study. To estimate F_ROH_, a percentage of homozygosity was calculated by summing ROH >1 Mb across the covered genome and dividing by the total base pairs represented in the SNP data obtained by calling SNPs from the Canadian and Iowan populations simultaneously. Runs of homozygosity were called using the --homozyg flag in Plink 1.9. To be considered ROH, segments had to be at least 1 Mb in length and have a maximum gap between SNPs of 500 Kb. However, several levels of stringency for other criteria were used to calculate ROH segments: Three sizes of window were examined (5, 10, and 20 SNPs) with 1, 2, and 3 heterozygotes per window allowed. These 6 levels of stringency were used to calculate Froh.

## 3. Results

### 3.1. Whole-Genome Sequencing and Variant Calling

After removing duplicate reads, whole-genome sequencing of the 12 individuals from Iowa produced an average of 217,503,897 (±131,486,905) reads per individual, out of which 96.27% were considered of high quality (quality score > 20) and retained for further analyses. Here, an average of 86% (±0.57) of the reads mapped to the reference Northern Pike genome. This produced an average depth of 26.26× that ranged from 8× to 49× presumably for differences in sample quality. In the case of the samples from the Canadian population, 86% of the reads were considered high quality, 63% of the high-quality reads were successfully aligned to the Northern Pike genome and a depth of 11.44× was obtained for the sequenced sections of the genome. Details on the sequence data of each individual from Iowa and the averages for the Iowa and Canadian populations are shown in Table 1.

Breadths of coverage at different depth thresholds are shown in Table 2. After aligning the reads of Muskellunge from Iowa to the Northern Pike genome, 80% of the bases were covered with a depth larger than 0×. Overall, 66% of the bases were covered with a depth of 10×, which was used as a threshold to be included in all downstream analyses. Also, 0.03% of the genome was covered at a depth higher than 1000×; potentially pointing at highly repetitive segments that showed issues with proper alignment and therefore were counted as the same segment [29].

**Figure 2:**
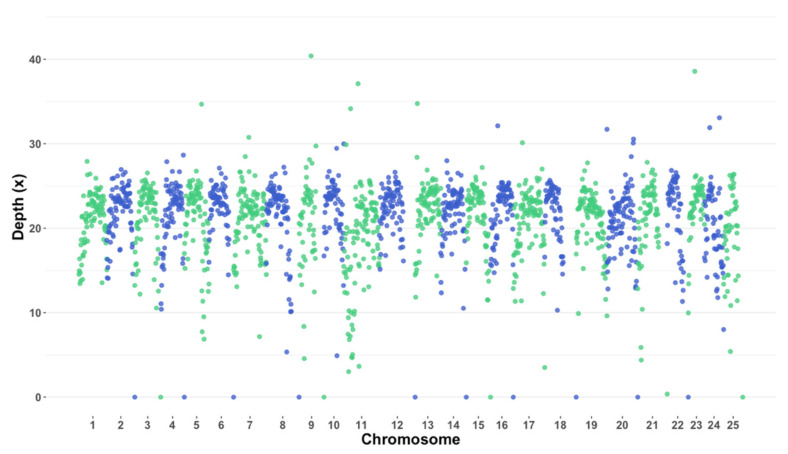
Average depth of coverage per megabase across all Iowa samples. Chromosomes vary in color for easy identification.

Figure 2 shows the average depth coverage per megabase across the twelve individuals from Iowa after the sequencing was aligned to the Northern Pike genome. The overall average depth across all 1 megabase windows was of 21.16× with a standard deviation of 4.44×. Depth of coverage was highest at chromosome 9, megabase 18.5, reaching a depth of 40.4×. Additional peaks were observed in chromosome 11 megabase 22 and chromosome 23 megabase 10 with depths of 37.1 and 38.6, respectively. Additionally, chromosomes 2, 3, 4, 6, 8, 9, 12, 14, 15, 16, 18, 20, 22 and 25 produced a depth of 0× on the last megabase. Finally, all chromosomes showed higher depth towards the centromere compared to the telomeric regions.

The variant calling pipeline produced three large sets of Single Nucleotide Polymorphisms (SNPs) that were used in further analyses. When variants were called for the twelve whole-genome sequenced samples from Iowa, a total of 36,627,942 biallelic SNPs were called out of which 8,218,039 were not monomorphic. Given the large number of SNPs, all SNPs that did not have a call rate of 100% were dropped, resulting in a final set of 7,886,471 SNPs that was used for all analyses involving only individuals from Iowa. The variant calling of Canadian samples produced 128,213 biallelic SNPs, out of which 16,059 were not biallelic.

The transitions/transversions ratio (Ti/Tv) was calculated for the two set of samples, and the results were of 1.09 and 1.29 for samples from Iowa and Canada, respectively. 

**Figure 3:**
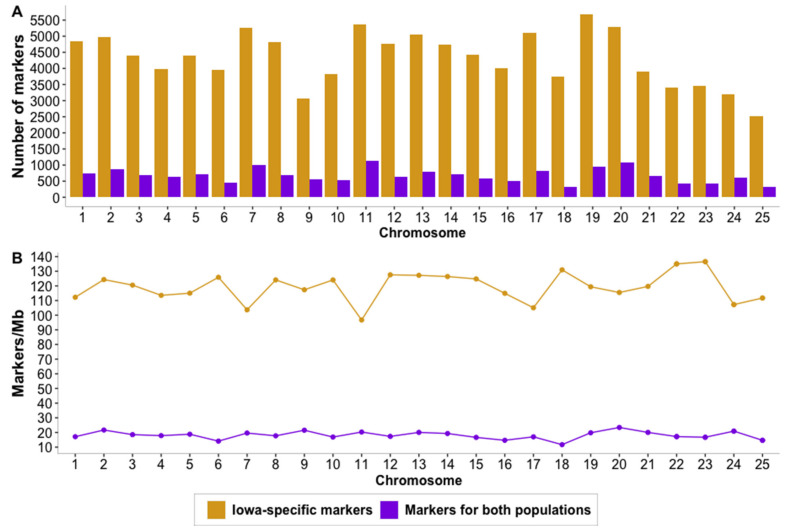
Distribution of single nucleotide polymorphisms (SNPs) by chromosome. (**A**). Average number of population-specific SNPs for the Iowan (yellow) and for both populations combined (purple). (**B**). Average density of population-specific SNPs per chromosome for Iowan (yellow) and for both populations combined (purple).

When combining the two Canadian and Iowan dataset, a total of 108,132 biallelic SNPs were called with 22,705 SNPs not being monomorphic. After retaining only SNPs with a call rate larger or equal to 90%, a total of 16,867 SNPs were kept for further analyses. The number of Iowa-specific biallelic SNPs and biallelic SNPs called for Iowa and Canada along with the density of SNPs per megabase are shown in Figure 3 panels A and B, respectively. As shown in Figure 3A, chromosome 19 showed the highest number of biallelic SNPs with 5675 SNPs, while chromosome 11 showed 1125 SNPs that were common between Canada and Iowa samples, being the chromosome with the most SNPs. On the other hand, chromosome 25 was the chromosome with the least number of SNPs in both cases with 2525 and 331 respectively. On average, 4162 and 644 SNPs per chromosome were called for the samples from Iowa and the combined samples with a standard deviation of 872.84 and 216.78, respectively. When looking at the density of SNPs per Mb as shown in Figure 3B, the chromosome with the highest density of SNPs was chromosome 23 with 136.54 SNPs per megabase when SNPs were called for the Iowan population only and chromosome 20 with 23.41 SNPs per megabase when SNPs were called for the Canadian and Iowan populations simultaneously. On average 120 SNPs per megabase were called for the Iowa population and 18 SNPs per megabase were called for the Canadian and Iowan populations combined with a standard deviation of 10.44 and 2.62 SNPs per megabase, respectively.

### 3.2. Population Stratification Analyses

**Figure 4:**
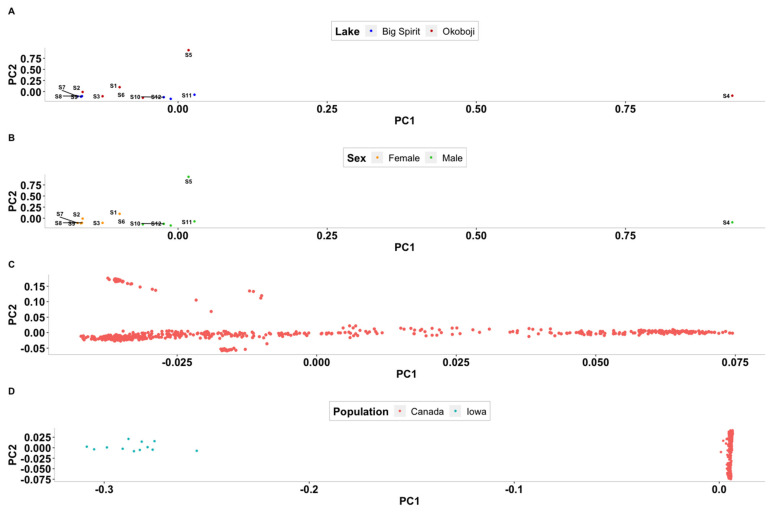
Principal component analysis (PCA) results. (**A**) Samples from Iowa colored by lake of origin. Big Spirit in blue and Okoboji in red. Principal components 1 and 2 account for 11.74% and 11.72% of the variance, respectively. (**B**) Samples from Iowa colored by sex. Females in orange and males in green. Principal components 1 and 2 account for 11.74% and 11.72% of the variance, respectively. (**C**) Samples from Canada. Principal components 1 and 2 account for 27.24 % and 20.58% of the variance, respectively. (**D**). Principal component analysis (PCA) results for Iowa and Canada populations combined. Principal components 1 and 2 account for 65.24% and 9.39% of the variance, respectively. PC1 and PC2 indicate principal component 1 and 2, respectively. Canada samples in red and Iowa samples in teal.

Principal Component Analysis (PCA) results for the Iowa and Canadian populations are shown in Figure 4. No clustering was observed when comparing Iowan samples (Figure 4A). Given that individuals were sampled at two different lakes that are known to be connected, these results were not surprising although it is important to note that individuals 4 and 5 did not cluster with the rest of samples. However, in Figure 4B a clustering by sex can be observed with the exception of individuals 4 and 5 that did not cluster with the other males. This clustering may indicate some level of genetic differences between individuals of the different sexes and not sex differences themselves. Figure 4C shows several clusters that were identified in the Canadian population which are likely related to what part of the water system they were sampled from. When PCA was performed on Iowa and Canada samples combined (Figure 4D), populations form Iowa and Canada clustered separately, indicating genetic differences between the two populations.

**Figure 5:**
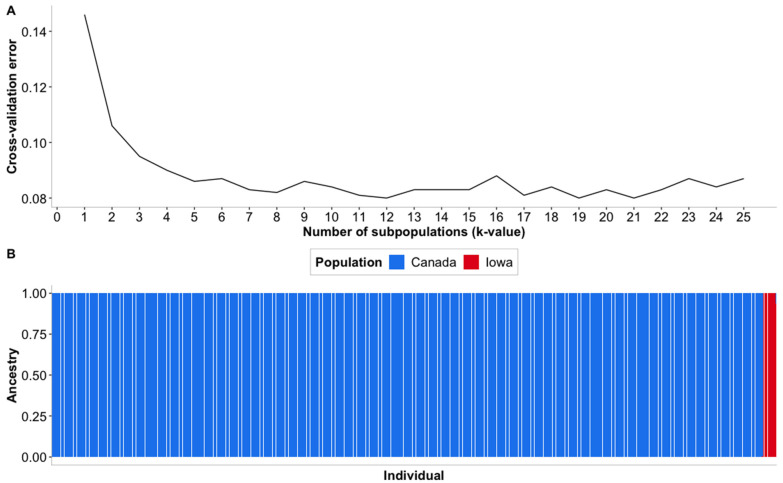
(**A**). Cross-validation error value for multiple subpopulation numbers. (**B**). Admixture plot for two subpopulations.

Admixture analyses confirmed the findings from PCA. Results from admixture analyses are shown in Figure 5. When both the Iowa and Canada populations were analyzed, the likely number of subpopulations found was between 12 and 19 as these numbers produced the lowest values for the cross-validation error (Figure 5A). Here, admixture detected clear differences between populations from Iowa and Canada. The differences between populations were so large that when the results of Admixture with k = 2 were plotted (Figure 5B), all 12 individuals from Iowa showed a composition >0.9 for the same subpopulation while all samples from Canada showed a composition of 1.0 for the second population. In a similar matter when ancestry estimations were plotted for k values of 12 and 19 (shown in Supplementary Figure S2 panels A and B, respectively) all 12 individuals from Iowa grouped in the same subpopulation with a composition >0.9 in both cases, illustrating the high degree of differentiation that exists between samples from Iowa and Canada.

### 3.3. Pooled Heterozygosity and Genome Wide Fst

**Figure 6:**
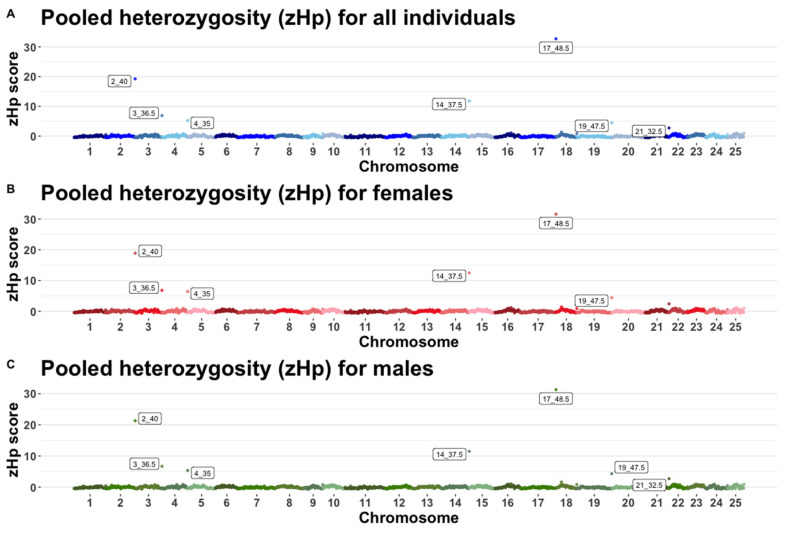
Mean pooled heterozygosity (Hp) values for 0.5 megabase windows with a 50% overlap for (**A**). All individuals (**B**). Females only. (**C**). Males only.

Regardless, the subgroup of the Iowa population that was considered for pooled heterozygosity (Hp) analyses (all individuals, females only and males only), the same windows were seen to be highly heterozygous in all cases, as shown in Figure 6. All these six windows showed normalized pooled heterozygosity scores that were more than three standard deviations from the mean and were therefore identified as outliers. Table 3 shows the six windows that had high heterozygosity. In total, 244 genes were found in these windows although only 53 have been previously annotated (Genes and coordinates shown in Supplemental Table S1). Given the small number of annotated genes found in these six windows, gene ontology analyses only showed one significantly enriched term, this being sensory perception of pain. Other enrichment terms found included sensory perception, positive regulation, or synaptic transmission and regulation of glial cell proliferation (Complete list of enriched GO terms related to Hp analyses shown in Supplementary Table S2).

Although PCA showed a clear clustering according to sex in the Iowan population, Fst analyses did not provide any insight on the differences. The highest mFst value found was of 0.09 and the overall Fst value between sexes was of 0.05 (Shown in Figure S1). Nonetheless, given that previous literature has shown that the Anti-Mullerian Hormone gene (*amh*) is responsible for sex determination in Northern Pike (*Esox lucius*), the genotypes of the individuals from the Iowa population were manually examined. As shown in Table 4, the genotype of 47 SNPs located on this gene were found to consistently differ between the 6 males and 6 females sampled. For these 47 SNPs, all males were heterozygous while all females were homozygous.

**Figure 7:**
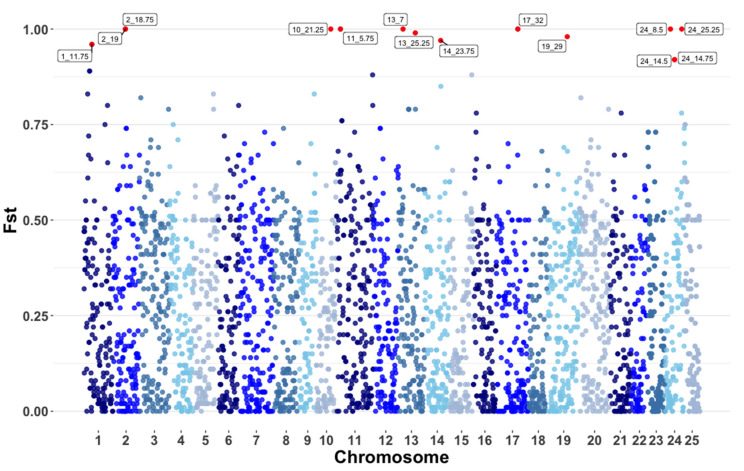
Mean Fst (mFst) values for 0.5 megabase windows with a 50% overlap contrasting the populations of Iowa and Canada. Chromosomes vary in color for easy identification.

As shown, population stratification analyses in Iowa and Canada have markedly different genetic backgrounds, and this is reinforced by the mFst values obtained for the comparison between both populations, where the overall Fst value was 0.24. Window-based Fst results are shown in Figure 7. In total, 14 windows produced a mFst value larger than 0.9 and 8 of these windows had an mFst value of 1, indicating that the majority of the SNPs in the window are fixed or almost fixed for opposite alleles. All windows that were deemed of interest after performing analyses of signatures of selection, had been sequenced at a depth that ranged from 17× to 32× and thus are considered as accurate results. This warrants a more in-depth analysis that might shed light on regions of the genome that are responsible for adaptation to the different specific environments. A total of 641 genes were identified in the 14 windows with mFst scores higher than 0.9. However, only 331 of these genes have been annotated and as in the case of Hp analyses, no statistically significant enriched terms were found (List of annotated genes found in mFst windows with score higher than 0.9 shown in Supplemental Table 3). Although not significant, several GO-terms associated with development and growth were enriched, these included negative regulation of developmental process, positive regulation of chondrocyte differentiation and positive regulation of cartilage development, among others (Complete list of enriched GO terms related to Fst shown in Supplemental Table S4). 

### 3.4. Inbreeding and Runs of Homozygosity (ROH)

**Figure 8:**
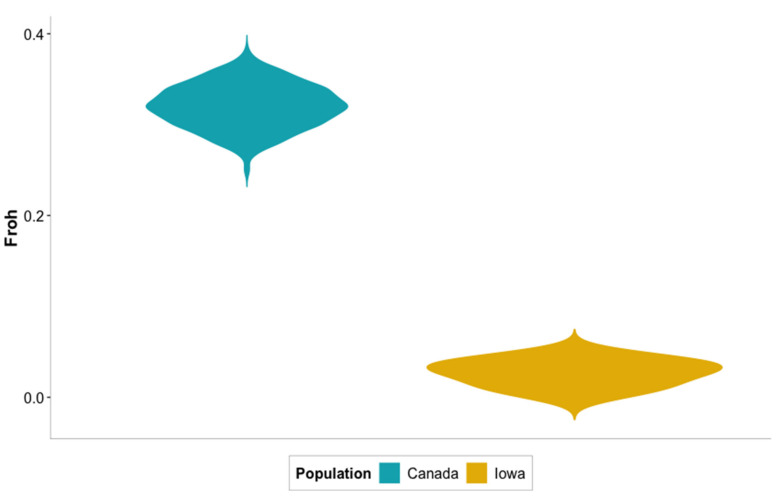
Distribution of inbreeding coefficients (Froh) for the populations of Iowa and Canada.

Inbreeding coefficients ranged from 0.00 to 0.44 depending on the level of stringency considered to call a segment as a run of homozygosity. Nevertheless, out of the six different stringency levels at which runs of homozygosity were analyzed, the level that was considered to produce the most realistic levels of inbreeding was with windows that included at least 20 SNPs while allowing a maximum of three heterozygotes. Individual details for the Iowa population and the average for the Canadian population are found in Table 5. On average, individuals from Iowa showed 3.5 ROH segments with a length of 36,699.20 Kb. The individual with the highest number of ROH segments was sample 9, a female from Big Spirit Lake with 7 segments of 50,042.8 Kb of length in average. In contrast, in sample 1, a female from Okoboji did not show any ROH segments. On average, females showed slightly higher number of ROH segments than males. However, these segments were approximately 2000 Kb shorter than in males. As shown in Figure 8, individuals from Canada showed a markedly higher level of inbreeding than samples from Iowa, being on average 0.32. Additionally, the Canadian population showed a slightly wider distribution of estimated inbreeding coefficients, ranging from 0.25 to 0.38 while the Iowa population shows a very short range from 0.00 to 0.05. The length of the segments in both populations is very similar, spanning about 6500 Kb. 

## 4. Discussion

### 4.1. Whole-Genome Sequencing, Alignment to Northern Pike Genome and Variant Calling

One of the main limitations of the present study is the absence of a reference genome for Muskellunge. Therefore, the traditional bioinformatics pipeline used in whole-genome sequencing analyses had to be adapted, to map the reads against the reference genome for Northern Pike which was available. Northern Pike is an esocid species closely related to Muskellunge with the same number of chromosomes (2*n* = 50). There is evidence that using a highly related species is a valid option in mammals [30], where Donkey (*Equus anus*) reads were aligned to the Horse (*Equus caballus*) genome and this approach has been used previously in Muskellunge [9]. The effectiveness of this approach in fish is reflected in the high percentage of reads that were correctly aligned to the Northern Pike genome (86%) and the breadth of coverage at a depth of at least 10× obtained after alignment of Muskellunge reads to the Northern Pike genome (66%). This being said, there are clear issues with alignment possibly due to differences between species that are reflected in the regions that show reading depths higher than 1000×. These reading depths can arise from copy number variations and/or chromosomal differences within species [31]. However, it is known that next generation sequencing has inherent issues with repetitive regions due to the short read-length seen in this technology [29] and therefore the exact cause cannot be determined. 

The Ti/Tv value refers to the ratio of transitions to transversions observed in the variants called. Transitions are variants within the same type of nucleotide while transversions are mutations from a pyrimidine to a purine or vice versa [32]. The Ti/Tv ratio observed in the datasets used in this research were in line with what has been reported in fish. While in mammals the Ti/Tv value is expected to be near 2.0 [32], values in fish have been observed to be lower, 1.28 for a closely related pike species [33], 1.49 in salmonids [34,35] and ranging from 0.28 to 1.49 in several teleost species [24,25,35]. While the increasing number of teleost species sequenced has confirmed this difference compared with mammals and the need to investigate its evolutionary meaning, this value can be also used as a quality parameter for the variant calling. This is of particular relevance in our work, as a closely related species was used as reference genome.

### 4.2. Population Stratification 

The PCA results reinforce the hypothesis that individuals from Iowa originate from the same strain. Even though individuals were caught at two different lakes, these lakes are interconnected, with fish from Spirit Lake being able to swim into Okoboji but not in reverse. Moreover, both lakes are stocked with fish from the same hatchery, where broodstock from the two lakes are mated and no pre-selection based on genetics is performed. Nonetheless, individuals s4 and s5 did not cluster with the rest of the samples from Iowa in Figure 4A,B. This may indicate that despite the lack of pre-selection of the broodstock, Iowan population showed a degree of genetic diversity. However, the low number of individuals sampled does not allow one to evaluate the degree of separation. The multiple different clusters seen in Figure 4C,D are consistent with the expected results of Rougemont et al. [9] where the fish were sampled from 22 different locations. Furthermore, admixture analyses confirm the large number of subpopulations seen in the Canadian samples as well as the marked difference seen between Iowa and Canada populations. It is important to note that no phenotypic information from the Canadian samples was available to contrast the Iowan and Canadian population and therefore the idea that there is differences related to specific environmental adaptations of the populations is based only on the existing genetic data. Therefore, phenotypic analyses should be implemented in the future to confirm results of genetic analyses. The original research determined that the number of subpopulations present in these samples was between 8 and 13, while our results indicate that the number lies between 12 and 19, these values could have changed since a different reference genome version was used in the studies. The clear separation between the Canadian samples and the 12 samples from Iowa in Figure 5A illustrates that the differences between Canadian and Iowan fish are larger than those between Canadian subpopulations. The results of both PCA and admixture analyses highlight the idea of both populations having adaptations to their specific environments that have caused them to diverge. Furthermore, these results support previous findings in that they suggest a number of genetically different populations throughout the geographical distribution of Muskellunge [36]. These differences are also likely to be enlarged due to populations having different origins given that Iowa Muskellunge originally descended from fish from Wisconsin [1,5] while fish from Canada descend from local broodstock [9].

### 4.3. Signatures of Selection and Inbreeding

Pooled heterozygosity revealed six windows of higher heterozygosity along the genome and no windows show high homozygosity independently of how the Iowa population was parsed. These 6 windows showed a high number of genes with an average of 40.6 genes per window. These results exemplify the low homozygosity estimated in the Iowa population and support the results of ROH analyses. Although the only significantly enriched GO term was related to perception of pain, several other GO terms found were related to perception and neurological processes like positive regulation of synaptic transmission, regulation of glial cell proliferation and positive regulation of glial cell proliferation. However, due to the small sample size, the interpretation of these GO terms has to be taken with caution.

Results of mFst back those of PCA and admixture analyses, showcasing the marked genetic differences between both populations. Similarly to Hp, the 14 windows that had mFst values above 0.9 contained a large number of genes, with an average of 46 genes per window. 

In teleost fish, sex determination is achieved through a wide variety of mechanisms that include genetic, environmental and social factors [37]. The clustering of sexes seen in Figure 4B indicates that possible genetic differences between the sexes exists. Previous research found the master sex-determining gene in Northern Pike is located in chromosome (i.e., linkage group) 24 [38]. This motivated us to perform a Fst analysis between sexes despite the low number of animals. Here, when allele frequencies were compared between males and females from Iowa, low mFst values across the genome were found, including chromosome 24. However, after thorough examination, the Anti-Mullerian Hormone gene (*amh*), which was deemed as the master sex determinant gene in Northern Pike is located in chromosome 8 on the newest reference genome for the species, which was used in the present research. As shown in Table 4, several SNPs distributed along the *amh* gene consistently show different genotypes in males and females. Although further analyses are needed for confirmation, these findings suggest that the *amh* gene may play a key role in sex determination in Muskellunge. 

Several windows were found to have mFst scores of 1 when the populations from Iowa and Canada were compared. This reinforces the findings of PCA and Admixture analyses and was expected since both populations have distinct origins and have been isolated from each other to the best of our knowledge. Previous research has shown the presence of private alleles in the majority of populations of esocids in the Great Lakes [3,39,40,41]. If the same is true for the Iowa and Canadian populations, private alleles to either of the populations could be responsible for the high mFst scores found when comparing them. Since the reference genome of *Esox lucius* is not thoroughly annotated many genes located in the windows with high mFst values were unannotated and thus caused gene ontology analyses to be unsuccessful. This being said, although not significant, a large number of enriched GO terms were related to growth and cellular differentiation, which highlights differences between both populations and perhaps their adaptation to the different environmental conditions present in their geographical locations. Additionally, after manual verification, several genes were found to be linked to congenital disorders and other fitness effects. Genes linked to congenital and behavioral disorders included dynein axonemal intermediate chain 1 [42], Rho GTPase Activating Protein 36 [43], ATPase Na+/K+ Transporting Subunit Alpha 3 [44] and 5-Hydroxytryptamine Receptor 2C [45] while genes related to fitness effects include genes such as Cysteine-Three-Histidine [46].

Given the type of genomic data available, runs of homozygosity were deemed the most appropriate method to estimate some level of inbreeding. However, given the arbitrary method in which stringency levels are set up to identify ROH segments [47], several thresholds were tried before reporting a final result. The small number of ROH segments detected at the chosen stringency level reinforce the results of pooled heterozygosity analyses given that none of these analyses showed highly homozygote regions. It also indicates that inbreeding depression does not represent an immediate concern for the Muskellunge population in Iowa. However, a larger number of individuals is needed to confirm these results. Although inbreeding may not pose a threat in the short-term for Iowa’s Muskie population, caution must be taken since previous research in Minnesota has found statistically detectable reductions in genetic diversity compared with the wild source population [3]. Therefore, it is paramount to implement measures that limit the loss of genetic diversity in the population, especially in the lakes where brood stock is routinely captured. As suggested by Miller et al [3], measures aimed at this purpose include increasing the frequency at which wild germplasm is collected and using larger numbers of adults as broodstock [3].

In the case of the Canadian population, the markedly higher inbreeding coefficient is in line with previous research, confirming that the genetic structure of these population represents bottlenecked subpopulations of the overall St. Lawrence River population. These bottleneck events could have been caused by the small number of founders from the populations used to stock these locations [9]. Given these results, attention should be paid to managing the genetic diversity within the different subpopulations since this is critical to support the genetic viability of native populations, as these allow for a set of diverse genetic resources for reintroduction of the species to lakes where populations have disappeared and the supplementation of other populations that show loss of genetic diversity [3]. Moreover, it has been shown that Muskellunge display a high degree of spatial genetic structure that show clearly subpopulations within each population. This could be due to geographic isolation or the known reproductive fidelity to spawning habitats that the species shows [48,49], which further supports these results. With this scenario, populations would differentiate from each other, giving rise to the distinct subpopulations found with stratification analyses. As a result, homozygosity would rise within each subpopulation [50], producing the inbreeding seen through Froh analyses. Interestingly, the length of the ROH segments seems to be similar in both populations, which may indicate that the inbreeding that produced the homozygosity happened at similar times. This notion is reinforced given that stocking in Iowa started in the 1960s [1,5] and Canada’s stocking began in 1951 [9]. If this assumption held to be true, the estimated higher levels of inbreeding in the Canadian population would indicate a higher rate of inbreeding in this population.

## 5. Conclusions

This genomic study is the first of its kind to focus on the Muskellunge population in Iowa. The results of the study provide the following conclusions:

Although special attention is needed to filter variants appropriately, using the genome of a closely related species (Northern Pike) as a reference for alignment is a valid approach to perform population genomic analyses when no existing reference genome is available.

- Despite genetic differentiation based on sex, no major locus has been detected.
- Muskellunge from Canada and Iowa represent two clearly distinct populations with different estimated rates of inbreeding.
- Inbreeding does not seem to be an immediate concern for Muskellunge in Iowa.
- Apparent isolation of subpopulations has caused levels of homozygosity to be higher in the Canadian Muskellunge population.

These results provide insight into the validity of using genomes of closely related species to perform genomic analyses of species that have no reference genome assembly available. Additionally, these approaches can be used to develop assessment procedures to monitor the long-term viability of the management practices of Muskellunge in US and Canada.

## Figures and Tables

**Table 1:** Depth of coverage for raw whole-genome sequence data for Iowa samples.

Sample #	Sex	Lake	Sequenced Reads (#) ^1^	Aligned Reads (%)	High-Quality Reads (%) ^2^	Depth (×) ^3^
1	Female	Okoboji	203,824,613	86.34	96.85	16.23
2	Female	Okoboji	315,700,163	86.16	96.78	24.93
3	Female	Okoboji	306,850,333	86.04	96.99	24.34
4	Male	Okoboji	99,981,043	87.30	96.22	8.26
5	Male	Okoboji	150,029,226	86.38	96.75	12.12
6	Male	Okoboji	538,733,400	87.32	96.44	41.53
7	Female	Big Spirit	438,773,328	86.29	95.68	49.98
8	Female	Big Spirit	538,733,400	85.62	95.29	47.41
9	Female	Big Spirit	510,088,829	85.62	94.77	47.41
10	Male	Big Spirit	157,555,467	86.76	96.56	14.34
11	Male	Big Spirit	140,430,988	86.73	96.41	12.81
12	Male	Big Spirit	177,235,006	85.92	96.56	15.8
Iowa Average	---	---	217,503,897	86.37	96.27	26.26
Canada Average	---	---	1,022,373	63.11	86.15	11.44 ^4^

^1^ Reads aligned to Northern Pike reference genome; ^2^ Quality score > 20; ^3^ Depth shown is prior to mapping quality filtering; ^4^ Depth was calculated for sequenced sections; # indicates the value presented is a count; × indicates the values presented are times sequenced.

**Table 2:** Average breadth of coverage across Iowa samples.

Depth Threshold	Bases above Threshold (#)	Percentage (%)
>0×	735,465,012	80.05
10×	607,490,936	66.12
20×	539,961,789	58.77
50×	21,395,310	2.33
100×	7,981,231	0.87
1000×	260,029	0.03

# Indicates the values presented are counts.

**Table 3:** Pooled heterozygosity values for individuals from Iowa.

Chromosome	Megabase ^1^	Minor Allele Counts ^2^	Major Allele Counts	Hp ^3^	ZHp ^4^
17	48.5	746	1366	0.0009	32.71
2	40.0	826	2702	0.0006	19.24
14	37.5	1622	3994	0.0004	11.77
3	36.5	2954	6190	0.0002	6.90
4	35.0	2605	9131	0.0002	5.19
19	47.5	4486	8906	0.0001	4.44

^1^ Number of minor alleles counted across SNPs found in the megabase; ^2^ Number of major alleles counted across SNPs found in the megabase; ^3^ Pooled heterozygosity; ^4.^ Normalized pooled heterozygosity values.

**Table 4:** SNPs located in the Anti-Mullerian Hormone gene (*amh*) for which male and female Muskellunge show distinct genotypes.

Marker Position in Chromosome 8 (bp)	Female Genotype ^1.^	Male Phenotype ^2.^
12874481	GG	CG
12874504	TT	CT
12874507	CC	TC
12874514	CC	TC
12874526	CC	AC
12874527	TT	AT
12874533	CC	TC
12874566	AA	TA
12874586	GG	AG
12874588	CC	TC
12874615	AA	CA
12874618	AA	GA
12874754	CC	GC
12874758	CC	TC
12874767	GG	AG
12874789	TT	CT
12874791	AA	GA
12874806	AA	CA
12874809	TT	CT
12874836	GG	AG
12874848	GG	CG
12874854	CC	TC
12874858	CC	TC
12874860	GG	AG
12874865	TT	CT
12874882	AA	TA
12874913	TT	AT
12874914	GG	AG
12874937	CC	TC
12875043	AA	GA
12875046	CC	AC
12875056	CC	TC
12875960	AA	GA
12875988	TT	GT
12875992	TT	GT
12876003	CC	TC
12876008	CC	TC
12876021	TT	AT
12876022	CC	TC
12876024	CC	AC
12876026	TT	AT
12876049	CC	AC
12876056	TT	AT
12876057	CC	TC
12876063	CC	GC
12876066	AA	TA
12876083	TT	GT

^1.^ All females showed the same genotype at these SNPs; ^2.^ All males showed the same genotype at these SNPs.

**Table 5:** Individual and average Froh scores.

ID	Sex	Lake	# ROH ^1^	Total Kb	Av. length (Kb) ^2^	Froh
S1	Female	Okoboji	0	0.00	0.00	0.00
S2	Female	Okoboji	2	12,622.20	6311.08	0.01
S3	Female	Okoboji	3	23,042.30	7680.75	0.03
S4	Male	Okoboji	2	12,730.70	6365.36	0.01
S5	Male	Okoboji	2	15,358.40	7679.21	0.02
S6	Male	Okoboji	4	24,561.20	6140.30	0.03
S7	Female	Big Spirit	5	36,351.40	7270.28	0.04
S8	Female	Big Spirit	5	36,499.60	7299.91	0.04
S9	Female	Big Spirit	7	50,042.80	7148.97	0.05
S10	Male	Big Spirit	4	31,859.10	7964.77	0.03
S11	Male	Big Spirit	3	21,129.30	7043.11	0.02
S12	Male	Big Spirit	5	36,699.20	7339.84	0.04
Canada average ^3^	--	--	46	294,087.37	6446.51	0.32

^1^ Number of segments considered runs of homozygosity; ^2^ Average length of ROH segments in kilobases; ^3^ Average for all Canadian samples.

## Data Availability

Whole Genome Sequence data produced for this research has been submitted to NCBI’s Sequence Read Archive under BioProject PRJNA695782. Link to data: https://www.ncbi.nlm.nih.gov/bioproject/?term=PRJNA695782 (accessed on 6 January 2021).

## Supplementary Materials

The following are available online at https://www.mdpi.com/article/10.3390/genes12071021/s1, Figure S1 Mean Fst (mFst) values for 0.5 megabase windows with a 50% overlap contrasting males and females in the Iowan population. Figure S2: A. Admixture analysis results for Muskellunge populations from Iowa and Canada with 12 assumed subpopulations. B. Admixture analysis result for Muskellunge populations from Iowa and Canada with 19 assumed subpopulations. Table S1. Annotated genes located in windows found significant in pooled heterozygosity analyses. Table S2. List of enriched GO terms related to Hp analyses. Table S3. Annotated genes located in windows found significant in mFst analyses. Table S4. List of enriched GO terms related to Fst analyses.
