# Supplementary material for: Signatures of Selection and Genomic Diversity of Muskellunge (Esox masquinongy) from Two Populations in North America
Source: Genes (Basel). 2021 Jun 30;12(7):1021. doi: 10.3390/genes12071021 (PMC8303616; doi:10.3390/genes12071021)
Supplement: Supplementary file 1 [file genes-12-01021-s001.zip › FigureS1.pdf]

Signatures of selection and genomic diversity of Muskellunge (*Esox masquinongy*) from two populations in North America.

Josue Chinchilla-Vargas[1*], Jonathan Meerbeek[2], Max F. Rothschild[1], Francesca Bertolini[3].
[1] Iowa State University, Department of Animal Science, Ames, Iowa, 50011.
[2] Iowa Department of Natural Resources, Spirit Lake Fish Hatchery, Spirit Lake, Iowa, 51360.
[3] National Institute of Aquatic Resources, Technical University of Denmark, 2800, KGs. Lyngby, Denmark.

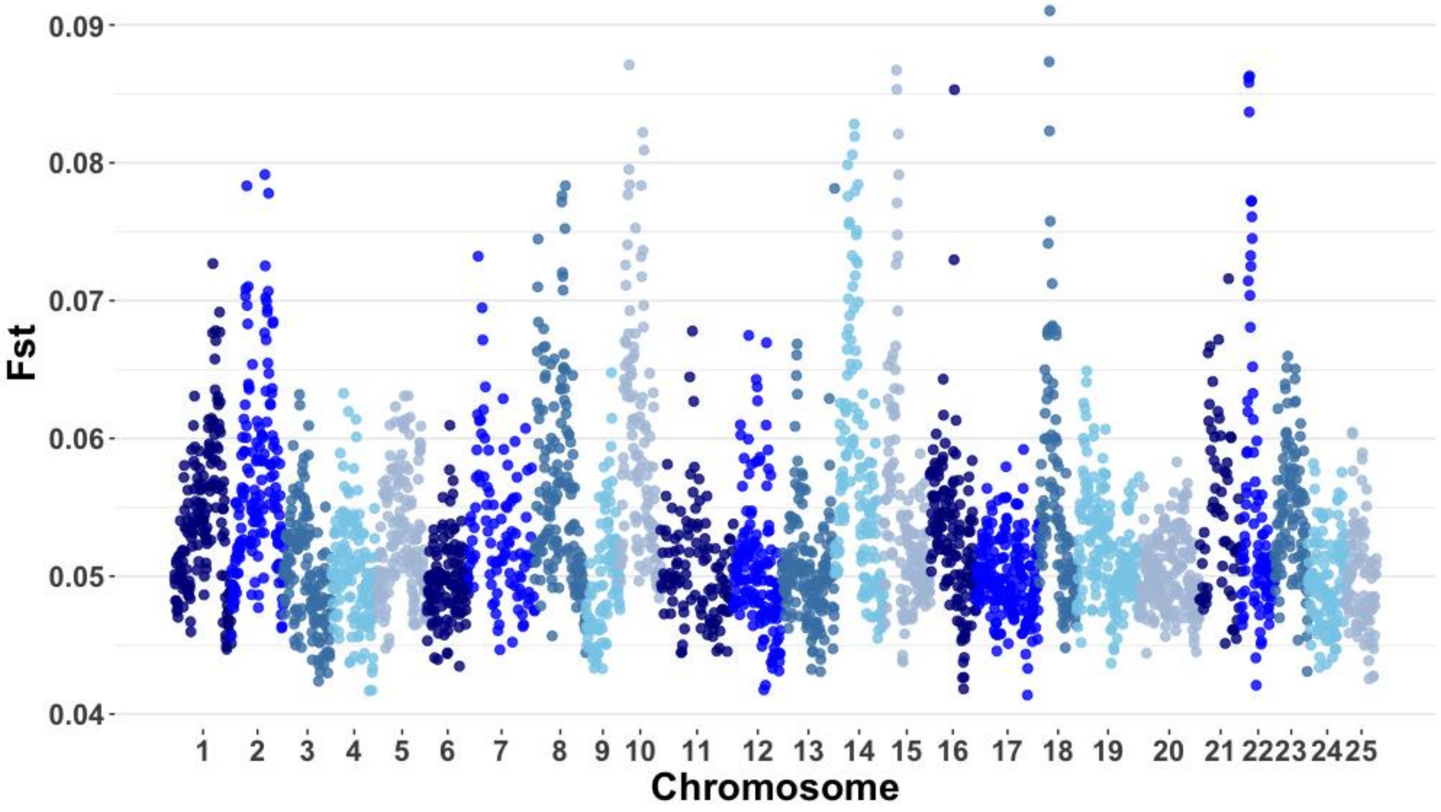

Figure S1 Mean Fst (mFst) values for 0.5 mega base windows with a 50% overlap contrasting males and females in the Iowan population.
