# Supplementary material for: Signatures of Selection and Genomic Diversity of Muskellunge (Esox masquinongy) from Two Populations in North America
Source: Genes (Basel). 2021 Jun 30;12(7):1021. doi: 10.3390/genes12071021 (PMC8303616; doi:10.3390/genes12071021)
Supplement: Supplementary file 1 [file genes-12-01021-s001.zip › FigureS2.pdf]

Signatures of selection and genomic diversity of Muskellunge (*Esox masquinongy*) from two populations in North America.

Josue Chinchilla-Vargas[1*], Jonathan Meerbeek[2], Max F. Rothschild[1], Francesca Bertolini[3].

[1] Iowa State University, Department of Animal Science, Ames, Iowa, 50011.

[2] Iowa Department of Natural Resources, Spirit Lake Fish Hatchery, Spirit Lake, Iowa, 51360.

[3] National Institute of Aquatic Resources, Technical University of Denmark, 2800, KGs. Lyngby, Denmark.

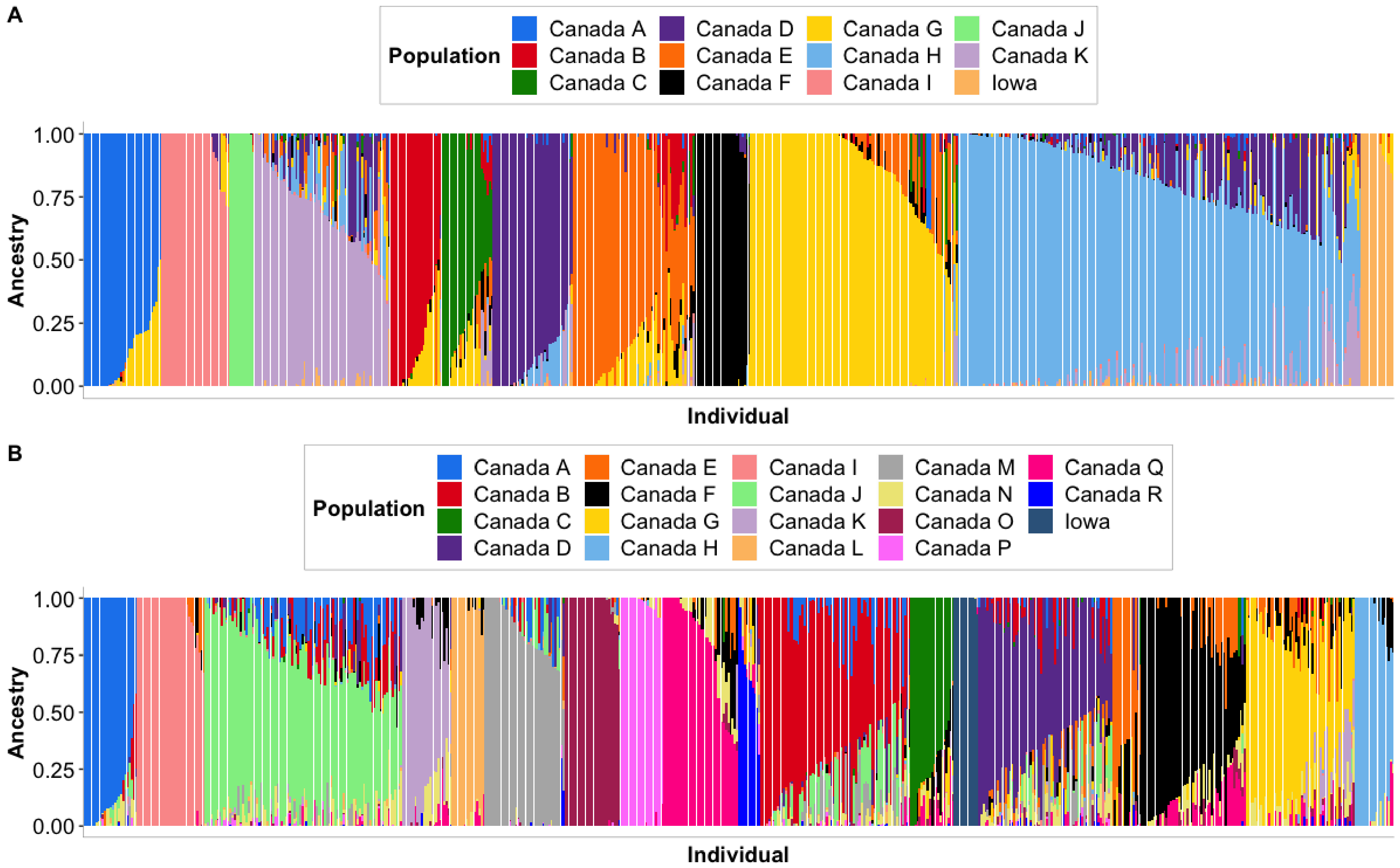

Supplementary Figure 1. A. Admixture analysis results for Muskellunge populations from Iowa and Canada with 12 assumed subpopulations. **B.** Admixture analysis result for Muskellunge populations from Iowa and Canada with 19 assumed subpopulations.
